# QT-Interval Prolongation and 1-Year Cardiac Death Among Patients Undergoing Hemodialysis

**DOI:** 10.1016/j.jacasi.2024.10.013

**Published:** 2025-01-07

**Authors:** Sho Sasaki, Kiichiro Fujisaki, Masato Nishimura, Toshiaki Nakano, Masanori Abe, Norio Hanafusa, Nobuhiko Joki

**Affiliations:** a Section of Education for Clinical Research, Kyoto University Hospital, Kyoto City, Kyoto, Japan; b Center for Innovative Research for Communities and Clinical Excellence (CiRC2LE), Fukushima Medical University, Fukushima, Japan; c Department of Nephrology, Iizuka Hospital, Fukuoka, Japan; d Tojinkai Hospital, Kyoto, Japan; e Department of Medical and Clinical Science, Graduate School of Medical Sciences, Kyushu University, Fukuoka, Japan; f Division of Nephrology, Hypertension and Endocrinology, Department of Medicine, Nihon University School of Medicine, Tokyo, Japan; g Department of Blood Purification, Tokyo Women’s Medical University, Tokyo, Japan; h Division of Nephrology, Toho University Ohashi Medical Center, Tokyo, Japan

**Keywords:** cardiac death, electrocardiography, hemodialysis, prognosis, QT interval

Cardiac events are the leading cause of death in patients undergoing hemodialysis (HD), accounting for approximately 30% to 40% of all deaths.^1^ However, the reasons for the high prevalence of cardiac death in patients undergoing HD are not fully understood.

We hypothesized that the high incident rate of cardiac death, including sudden cardiac death (SCD), in HD patients is caused by fatal arrhythmias resulting from prolonged QT intervals. Accordingly, we analyzed the association between QT intervals and cardiac death within 1 year using a nationwide Japanese data set of patients undergoing HD to verify this hypothesis.

This retrospective cohort study used the JRDR (Japanese Society for Dialysis Therapy and Renal Data Registry) for 2019 and 2020.^2^ The Medical Ethics Committee of the Japanese Society of Dialysis Therapy approved the protocol for this study (No.59).

We excluded all patients with missing QT intervals, with coexisting chronic atrial fibrillation, age <18 years, on dialysis <3 times/week, undergoing peritoneal dialysis, and undergoing home HD. Furthermore, patients were defined as outliers and excluded from the analysis if they had measurements for any of the following variables that exceeded 3 SDs from the mean: corrected QT interval (QTc), serum calcium, serum phosphorus, serum parathyroid hormone, serum magnesium, serum ferritin, serum iron, serum transferrin saturation, serum C-reactive protein, dialysis vintage, and heart rate.

The exposure was set as QT-interval prolongation. The QT and RR intervals were extracted from the most recent ECG data in the 2019 data set, and the QTc was calculated using the formula proposed by Bazett et al.^3^ This study defined QTc prolongation as QTc exceeding 500 ms, and it was used as a categorical variable for outcome assessment.

The primary outcome was the annual incidence rate of cardiac death, which was defined as death caused by heart failure, pulmonary edema, acute myocardial infarction, acute coronary syndrome, or arrhythmia. The secondary outcomes were the incidence of all-cause death and new cardiovascular events (CVEs). New CVEs were measured over a 1-year period, with eligibility limited to patients who had no history of CVEs in the data from the previous year.

We conducted a descriptive analysis of the patient characteristics. Univariable and multivariable analyses were performed using logistic regression models, with cardiac death, all-cause death, and new CVEs as outcome variables and QT-interval prolongation and QT interval as explanatory variables. In the multivariable analysis, age, sex, logged dialysis vintage, predialysis serum calcium and phosphorus levels, predialysis serum iron levels, coexisting diabetes, prior CVD, predialysis C-reactive protein level, and predialysis mean arterial pressure were adjusted. Logistic regression analysis (with cardiac death and new CVEs as outcomes) and inverse probability of censoring weighted analysis considering patients with noncardiac death as dropouts caused by factors other than the outcome were performed. Restricted cubic spline with 3 knots and population attribution fraction (PAF) were calculated based on multivariable models.

Analyses were performed using a 2-tailed alpha of 0.05.

## Results

A total of 197,401 patients met the inclusion criteria and were included for analysis. Among the participants included, 67,467 of 197,401 (34.2%) were women, 80,948 of 197,401 (41.0%) were patients with ESRD caused by diabetes, and 46,739 of 197,401 (23.7%) had a history of CVD. The mean age was 69.8 ± 12.4 years, the mean dialysis vintage was 66.0 months (Q1-Q3: 29.0-128.0 months), and the mean QTc was 450.6 ± 30.4. The number of patients with QTc >500 ms was 12,404 of 197,401 (6.3%).

The incidence rates of cardiac death, cardiovascular events, and death were 1.70% (3,365 of 197,401), 7.49% (10,119 of 135,108), and 7.28% (14,361 of 197,401), respectively. In the univariable analysis, the ORs for QTc >500 ms showed a statistically significant association with cardiac death (OR: 2.92; 95% CI: 2.32-3.66; *P* < 0.001), new CVEs (OR: 1.81; 95% CI: 1.48-2.21; *P* < 0.001), and all-cause death (OR: 2.13; 95% CI: 2.02-2.25; *P* < 0.001). Weighted multivariable analysis using inverse probability of censoring weighted for competing risks in cardiac death and new CVEs revealed statistically significant ORs for QTc >500 ms: cardiac death, OR: 2.09 (95% CI: 1.66-2.64; *P* < 0.001); new CVEs, OR: 1.64 (95% CI: 1.33-2.03; *P* < 0.001); and all-cause death, OR: 1.64 (95% CI: 1.55-1.74; *P* < 0.001).

**Figure 1:**
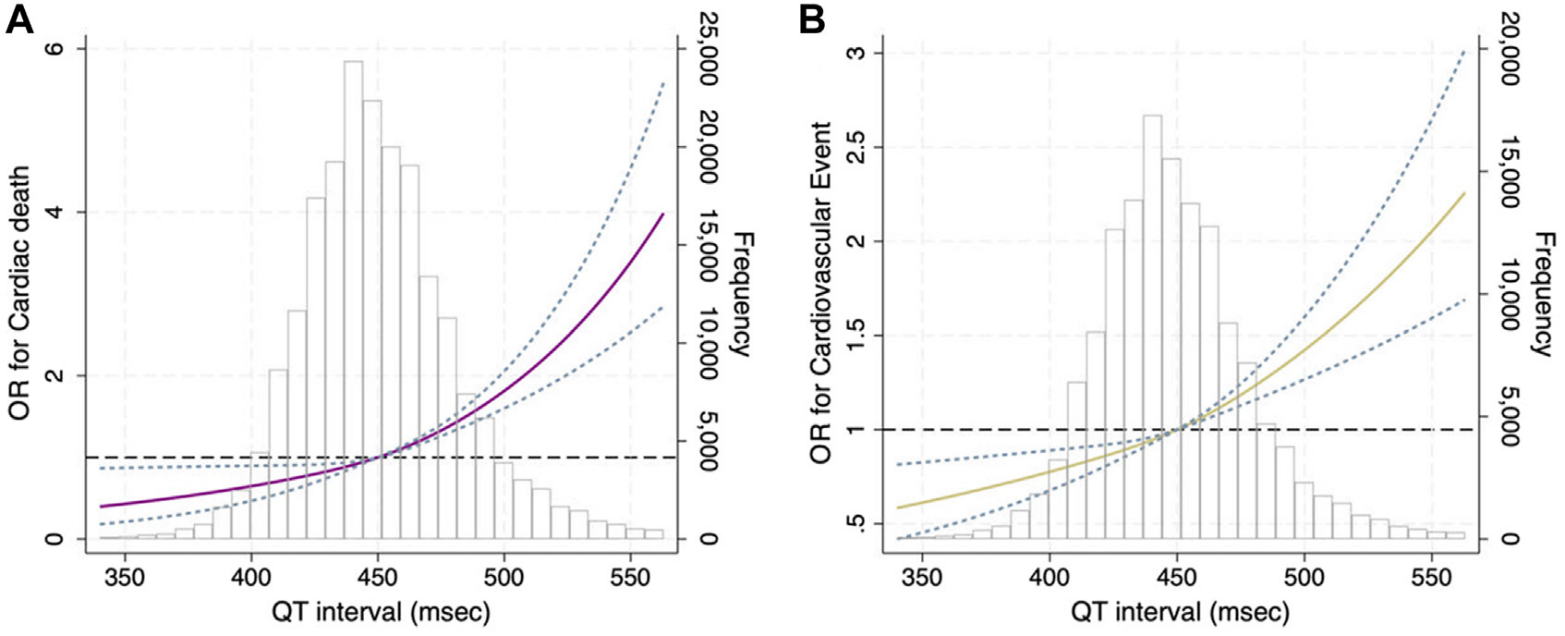
The Association Between QT Interval and Cardiac Death, New Cardiovascular Events In the restrictive cubic spline, the adjusted ORs for cardiac death (A) and new cardiovascular events (B) increased with prolonged corrected QT interval.

Figure 1 shows the results of restricted cubic spline. In the restrictive cubic spline, the adjusted ORs for cardiac death and CVEs were increased with prolonged QTc.

The adjusted QTc prolongation PAF for outcomes within 1 year was 6.32% for cardiac death (95% CI: 3.83-8.74; *P* < 0.001), 2.33% for new CVEs (95% CI: 1.25-3.41; *P* < 0.001), and 3.82% for all-cause death (95% CI: 3.31-4.32; *P* < 0. 001).

The results of this study demonstrated a statistically significant positive association between QT-interval prolongation and cardiovascular mortality within 1 year. Moreover, a prolonged QT interval was similarly associated with CVEs and 1-year all-cause mortality.

A previous study showed that significant differences between predialysis potassium concentrations and potassium concentrations in the dialysate and between predialysis calcium concentrations and calcium concentrations in the dialysate^3^ are associated with increased emergency department visits and death. Based on these findings, the association between QT-interval prolongation and cardiac death observed in this study may be explained by how patients experience fatal arrhythmias and SCD caused by dynamic QT-interval prolongation associated with dialysis therapy. The association between QT-interval prolongation and short-term cardiac prognosis within 1 year may be more reminiscent of arrhythmic death.

It is plausible that a prolonged QT interval is associated with cardiac outcomes such as SCD via fatal arrhythmias. However, our study also showed a strong correlation between a prolonged QT interval and noncardiac death. Although, common complications of HD patients include cirrhosis, sepsis, systemic lupus erythematosus, cerebrovascular disease, and chronic obstructive pulmonary disease, it is well known that QT time prolongation is often observed in these patients. Considering these findings, it is not surprising that QT-interval prolongation was associated with noncardiac and cardiac deaths in this study. In other words, an association is likely to be observed where QT-interval prolongation is present with these diseases; however, the cause of death may not be fatal arrhythmia leading to cardiac death but noncardiac death resulting from the respective pathological condition. In addition, antibiotics and other drugs used to treat these pathologies can prolong the QT interval.

To date, QT-interval prolongation has been reported in patients undergoing HD in various studies^4^ and has been validated in small observational studies to be associated with mortality. We believe that accumulating validation results using highly reproducible data, such as those from our study, could expedite discussions on the necessity of measuring QT intervals on ECGs, their utilization, and the effect of QT prolongation correction on prognosis in clinical practice. Our study’s PAF of cardiac death was estimated to be approximately 6.6%. A PAF of 6.6% can be interpreted as a potential 6.6% reduction in cardiac death in the overall population if all QT prolongations (>500 ms) are corrected.

This study has some limitations. First, unmeasured confounding factors may have affected our results. Second, measurement errors may have introduced bias into the study. Third, the exclusion of patients with missing QT intervals from the analysis may have been a source of bias.

This study provides evidence of a positive association between QT-interval prolongation and cardiovascular death in patients undergoing HD. These findings indicate that the QT interval could serve as a potential indicator for improving the prognosis of patients undergoing HD.

## Funding Support and Author Disclosures

The results in the current study were derived from the analysis of data provided by the JSDT as a study by public competition. However, all interpretations and conclusions in the study are on the responsibility of the authors, not the JSDT. English editing of this article was supported by the Japan Society for the Promotion of Science KAKENHI Grant Number 23K16353. The authors have reported that they have no relationships relevant to the contents of this paper to disclose.
